# Retrieval of Entrapped Broken Guide Wire in the Femoral Head and Hip Joint Using Alligator Forceps: A Case Report and Technique Note

**DOI:** 10.1155/cro/1400908

**Published:** 2025-11-10

**Authors:** Kuan Kai Tung, Yuan-Shao Chen, Yu-Min Lin, Cheng Chi Wang, Kun-Hui Chen, Chung Yuh Tzeng

**Affiliations:** Department of Orthopedics, Taichung Veterans General Hospital, Taichung, Taiwan

**Keywords:** core decompression, entrapped broken guide wire, guide wire failure, reamer

## Abstract

The guide wire plays a crucial role in core decompression procedures for avascular necrosis of the femoral head and fixation of femoral neck fractures using dynamic hip screws (DHSs) or cannulated screws. Although guide wire breakage during surgery is extremely rare, there have been instances where the distal end of the guide wire has fractured and becomes trapped within the fractured femoral head or hip joint. This can occur due to technical errors or poor quality of the guide wire used. The removal of the broken guide wire from the femoral head presents a challenging and complex task that requires precision and efficiency. In this report, we present a safe and efficient method to remove a broken guide wire entrapped within the femoral head and hip joint using alligator forceps. Under the guidance of an image intensifier, the broken guide wire was successfully extracted using this specialized instrument. By sharing this rare case, our goal is to raise awareness among surgeons about the possible complications associated with core decompression procedures and to highlight the importance of preventive measures. Furthermore, we introduce a reliable and alternative technique for removing broken guide wires, providing surgeons with an easier and more efficient approach.

## 1. Introduction

Guide wire breakage is a rare occurrence during orthopedic surgical procedures, with a recent increase in reported cases involving hip fractures [1]. However, cases of broken guide wires in core decompression procedures are rarely reported. Removing a broken guide wire in these situations presents a challenging task for orthopedic surgeons. In this report, we present a case of a broken guide wire in a patient who underwent core decompression for avascular necrosis of the femoral head. The broken guide wire was entrapped within the hip joint, and the femoral head was successfully removed with alligator forceps. This case report is aimed at providing orthopedic surgeons with a safe and effective method for removing broken guide wires encountered during similar surgical procedures.

## 2. Case Report

**Figure 1:**
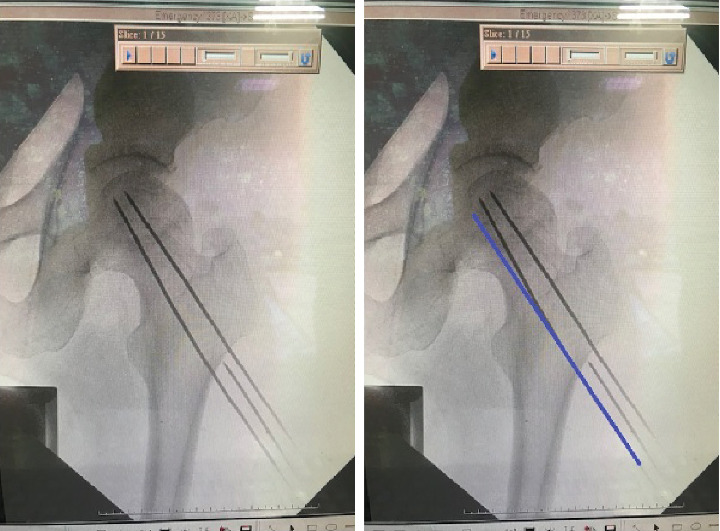
Three guide wires were attempted to be placed into the left femoral head under image intensifier guidance.

**Figure 2:**
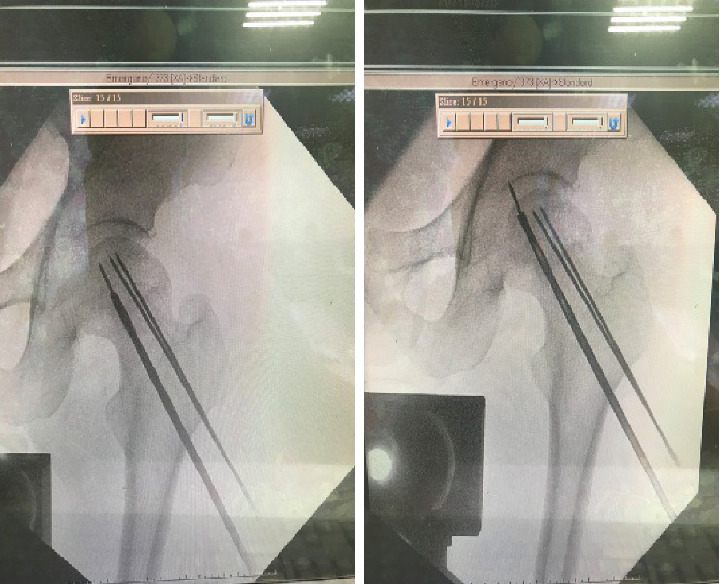
The broken guide wire was pushed into the hip joint after protrusion of the femoral head and engaged in the hip joint.

A 54-year-old male patient with avascular necrosis of the femoral head underwent core decompression of the left femoral head. During the procedure, three guide wires were attempted to be placed into the left femoral head under image intensifier guidance (Figure 1). Three 2-mm threaded guide pins were inserted into the femoral neck and head until their tips reached about 0.5 cm from the subchondral region. Then, the cannulated drill was used to ream the femoral head along with the guide wires to enlarge the bone tunnel to decompress the bone marrow pressure of the femoral head. After high resistance reaming through the hard tissue, the reamer suddenly passed through easily. When checking under the image intensifier, it was observed that the guide pin was broken. Unfortunately, a broken guide wire was noted after the first cannulated drilling during the operation. First, the removal of the broken guide was attempted with a cannulated drill 4.5 mm in diameter, but in vain. The broken guide wire was partially pushed into the hip joint after penetration of the femoral head and engaged in the hip joint (Figure 2).

## 3. Technique Note

**Figure 3:**
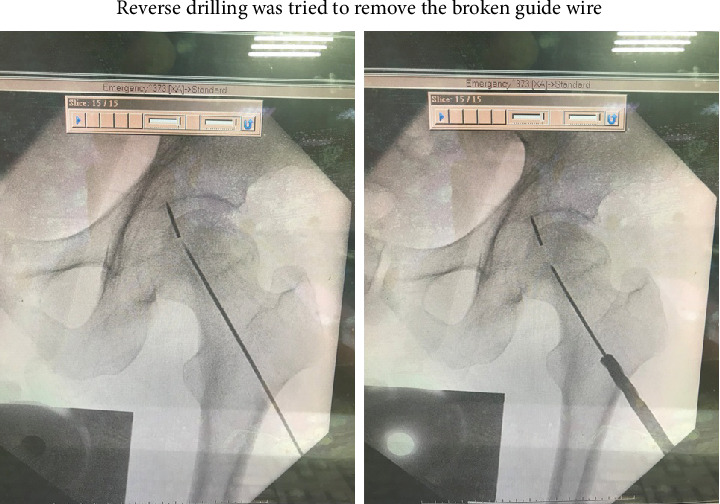
Reverse drilling was tried to remove the broken guide wire, but in vain.

**Figure 4:**
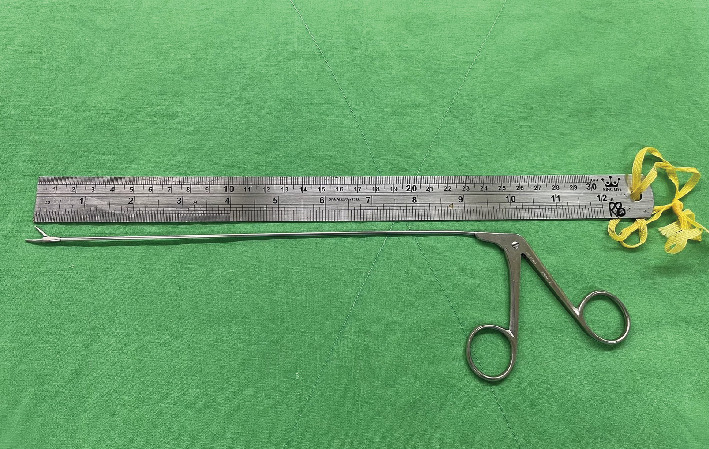
Alligator forceps (ELM).

**Figure 5:**
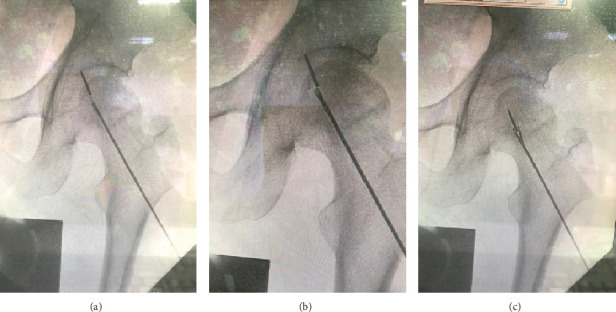
The tip of the broken guide wire was securely grasped by the alligator forceps under fluoroscopic guidance.

The new guide wire was put into the previous drilled tract as a new guide to reach the broken fragment of guide wire. Then, a 4.5-mm cannulated drill (AO, synthesis) was used to drill a new tract that can reach the broken end of the K-pin (Figure 3). Then, the alligator forceps (shaft length 8.5 inches, jars width 8mm) was put into the drilled tract smoothly. Opening the mouth of the alligator forceps and grasping the tip of the broken K-pin was tried under the fluoroscope guidance of the image intensifier. The broken guide wire, which had become lodged in the hip joint, was successfully grasped and removed using alligator forceps (ELM) (Figure 4). The tip of the broken guide wire was securely grasped by the cup forceps under fluoroscopic guidance (Figure 5). The procedure was carried out with no other complications, and the patient showed excellent tolerance and recovery postsurgery.

## 4. Discussion

Technical errors during intramedullary nail insertion have been reported since removing a broken guide wire from the hip joint presents a challenge for orthopedic surgeons, particularly when the wire is lodged deep within the joint or pelvic cavity [2, 3]. The primary factor leading to guide wire breakage is the bending of the wire, often resulting from aggressive reaming techniques and improper insertion. Inexperienced surgeons may overlook the importance of avoiding excessive force during reaming and maintaining vigilance throughout the procedure. Additionally, the quality of the guide wire itself can contribute to breakage, especially when reused. To prevent adverse events associated with guide wire placement and reaming, it is crucial to adhere to the recommended guidelines and best practices [2]. During the drilling and screw insertion process, it is imperative to use imaging technology to verify the position of guide wires. It is essential to discard guide wires at the end of the procedure, even if they appear to be undamaged. Reusing guide wires should be strictly avoided, and manufacturers should be encouraged to clearly label guide wires as single-use items. Furthermore, intraoperative cleaning of the cannulation reamer is crucial to prevent debris accumulation and minimize associated risks.

The primary contributing factor to the breakage of the guide wire in this case was the excessive bending of one guide wire, as observed in Figure 1, indicating a lack of proper alignment during the guide wire insertion process. The reason why the inferior pin was bent in the proximal femur of this patient might be caused by bending of the guide pin tip at the entrance of the hard lateral cortex of the proximal femur while drilling, which was ignored by the operator. The blended guide wire should be replaced by a new nonbending guide wire to prevent this complication next time. It should be kept in mind that the alignment of the K-pin is crucial before cannulated reaming to prevent breakage of the K-pin during cannulated reaming. It is also important that when the reaming is not smooth, the guide pin and reamer device should be checked by the image intensifier. Repeated use of guide wires can lead to deformation, loss of torsional strength, and reduced bending strength. The bending of guide wires may result from suboptimal surgical technique or the utilization of previously used guide wires in an effort to achieve precise placement within the femoral head. Such guide wires, when reused, are more prone to bending during insertion due to diminished strength. Consequently, the subsequent passage of a more rigid drill or reamer over the bent wire can result in jamming and eccentric loading, which compromises the integrity of the procedure [3].

The primary contributing factor to the breakage of the guide wire was the excessive bending observed in Figure 1, indicating a lack of proper alignment during the guide pin insertion process. In an attempt to retrieve the broken guide wire, a cannulated drill with reverse reaming was first tried to remove the broken guide wire. Regrettably, this maneuver resulted in the guide wire being inadvertently pushed into the hip joint and becoming trapped along the periphery of the femoral head. In this complex scenario, identifying an appropriate approach for the extraction of the distal broken pin presented a formidable challenge. Using alligator forceps under the precise guidance of the image intensifier, the broken guide wire was skillfully extracted from the affected region.

Several methodologies have been described in the literature to address the issue of broken guide wires, offering alternative approaches for consideration. Peivandi et al. [4] used a 2-mm cannulated drill bit for the successful removal of the broken guide wire in patients who underwent operative treatment for intertrochanteric fractures of the femur. Zhu et al. [5] report their way to remove the broken guide wire using a cannulated drill after a curette failure to remove it. They attempted to extract the broken part by utilizing a cannulated drill. The reaming process involved slowly rotating the cannulated drill around the threaded K-wire until the reamer fully engaged the target area under the guidance of the image intensifier. The cannulated drill was employed to clear the bone tunnel. Eventually, the broken end of the wire, mixed with blood and bone, was successfully removed. It was horrible and troublesome when broken guide occurred during operation. According to our previously clinical experience, this unfortunate situation with a broken guide wire happened twice before, it is lucky that those two situations were solved smoothly by aid of reverse drilling of the cannulated drill then. The broken guide wire which was retained in the femoral head without penetration was successfully removed using the Zhu's method. This case is more complicated due to failure of the removal of the broken guide wire with Zhu's method initially; eventually, the broken guide wire was pushed out of the femoral head and trapped within the femoral head and hip joint. It is simply too lucky to remove the broken guide wire with the aid of alligator forceps when the broken guide wire was partially remained with the femoral head. There is another case report to remove the broken guide wire with intrapelvic protrusion by Mishra and Gautam [6]. The surgeons opted for an open method, specifically the Watson-Jones approach, to create a window over the anterior cortex from the femoral neck. This allowed them to remove the broken fragment of the neck. Additionally, another window was created to extract the intrapelvic protrusion fragment of the broken guide wire using a plier. We have thought about the way to use an open method to remove the broken guide wire, but it is too invasive and traumatic in the condition of this case, especially in core decompression.

The utilization of an arthroscopic grasper for the extraction of a broken guide wire has previously been documented by Fard et al. [7]. If the broken guide wire still partially remains within the femoral head, using the alligator forcep removal method is a simpler procedure. Similar to their approach, our technique involved a direct lateral approach with minimal incisions.

The technique presented in this technical note is less invasive and technically simple. When the guide wire is properly positioned, we provide a valuable approach for removing broken guide wire from the hip joint. Surgeons should be aware of the potential complications associated with guide wire breakage and the importance of choosing appropriate retrieval methods. Different broken guide wire positions may need different retrieval methods; in similar situation, the simpler and minimally invasive method is the better method.

## Data Availability

The data are not publicly available due to privacy or ethical restrictions.
